# Syndecan-1 Expression Is Increased in the Aortic Wall of Patients with Type 2 Diabetes but Is Unrelated to Elevated Fasting Plasma Glucagon-Like Peptide-1

**DOI:** 10.3390/biomedicines9060697

**Published:** 2021-06-20

**Authors:** Stelia Ntika, Linda M. Tracy, Anders Franco-Cereceda, Hanna M. Björck, Camilla Krizhanovskii

**Affiliations:** 1 Department of Research, Södertälje Hospital, Södertälje, 152 86 Stockholm, Sweden; lindamtracy@gmail.com (L.M.T.); camilla.krizhanovskii@sll.se (C.K.); 2 Cardiothoracic Surgery Unit, Department of Molecular Medicine and Surgery, Karolinska Institutet & Karolinska University Hospital, 171 76 Stockholm, Sweden; anders.franco-cereceda@ki.se; 3 Cardiovascular Medicine Unit, Center for Molecular Medicine, Department of Medicine, Karolinska Institutet, Karolinska University Hospital, 171 76 Stockholm, Sweden; hanna.bjorck@ki.se

**Keywords:** thoracic aortic aneurysm, type 2 diabetes, adventitia, syndecan-1, glucagon-like peptide-1

## Abstract

A reduced prevalence of a thoracic aortic aneurysm (thoracic AA) is observed in type 2 diabetes (T2D). Glucagon-like peptide-1 (GLP-1)/GLP-1-based anti-diabetic therapy has indicated protective effects in thoracic AA and regulates the processes controlling the vascular tissue expression of Syndecan-1 (Sdc-1). Sdc-1 expression on macrophages infiltrating the aortic tissue contributes to a counter-regulatory response to thoracic AA formation in animal models through the interplay with inflammation/proteolytic activity. We hypothesized that elevated fasting plasma GLP-1 (fpGLP-1) increases the aortic Sdc-1 expression in T2D, which may contribute to a reduced prevalence of thoracic AA. Consequently, we determined whether T2D/thoracic AA associates with an altered Sdc-1 expression in the aortic tissue and the possible associations with fpGLP-1 and inflammation/proteolytic activity. From a cohort of surgical patients with an aortic valve pathology, we compared different disease groups (T2D/thoracic AA) with the same sub-cohort group of controls (patients without T2D and thoracic AA). The MMP-2 activity and Sdc-1, GLP-1R and CD68 expression were analyzed in the aortic tissue. GLP-1, Sdc-1 and cytokines were analyzed in the plasma. The aortic Sdc-1 expression was increased in T2D patients but did not correlate with fpGLP-1. Thoracic AA was associated with an increased aortic expression of Sdc-1 and the macrophage marker CD68. CD68 was not detected in T2D. In conclusion, an increased aortic Sdc-1 expression may contribute to a reduced prevalence of thoracic AA in T2D.

## 1. Introduction

A thoracic aortic aneurysm (thoracic AA) is increasing in prevalence and although it is less common than an abdominal aortic aneurysm (abdominal AA), it is more lethal; no screening programs are available [1] and treatment options are limited to surgical interventions. The most common form of thoracic AA occurs in the ascending aorta (ascending AA), which is the section of the aorta closest to the heart. Other forms of thoracic AA occur in the aortic arch and the descending aorta [2]. Surgical intervention in thoracic AA is needed before the ascending aorta reaches 4.5–5.5 cm, depending on the growth rate, possible risk factors, concomitant cardiac surgery, genetics and others [3,4]. The search for pharmaceutical agents and novel pharmaceutical targets for the prevention of an ascending AA is thus highly needed and has been fueled by the reduced prevalence of thoracic AA in patients with type 2 diabetes (T2D) [5,6], possibly related to the anti-diabetic therapy. Indeed, studies in animal models indicate that anti-diabetic incretin therapy, including glucagon-like peptide-1 (GLP-1) analogues and dipeptidyl peptidase-4 (DPP-4) inhibitors, may exert protective effects on ascending AA formation through anti-inflammatory and anti-oxidant effects, reduced intimal thickening, decreased matrix metalloproteinase-2 (MMP-2) and MMP-9 production and the suppression of macrophage infiltration [7,8,9,10,11,12].

Growing evidence supports an outside-in model where vascular inflammation is initiated in the adventitia, the outermost layer of the aortic wall [13,14,15]. Specifically, according to this model, exogenous cell types including macrophages and lymphocytes populate the adventitia, ultimately resulting in an increased local expression of cytokines and growth factors. This, in turn, may lead to an inflammatory response that propagates inward from the adventitia towards the media layer [16,17], causing medial degradation by MMPs, smooth muscle cell (SMC) loss and de-differentiation [13,15,18,19,20].

The cell surface proteoglycan syndecan-1 (Sdc-1) is a regulator of inflammation with a dual role in MMP activity, first as a regulator of the proteolytic activity and second as a substrate of proteases. It is mainly expressed on the surface of epithelial cells and non-circulating plasma cells but may be induced also in several other cell types including macrophages and SMCs. Sdc-1 may be proteolytically cleaved and shed from the cell surface by different MMPs such as MMP-2 and MMP-9 [21,22] in a process termed shedding. Shedding can be observed as a dramatic increase in the plasma concentrations of Sdc-1 ectodomains and occurs in response to different stimuli, e.g., inflammation, proteolytic activity and oxidative stress [23]. The aortic Sdc-1 expression protects from an abdominal AA formation in experimental models [24] where Sdc-1 knockout is associated with a reduced expression of the SMC differentiation markers and upregulated cytokine expression [25]. Its protective role is further indicated by the induction of Sdc-1 on infiltrating macrophages as a response to aneurysm formation where it provides an important counterbalance to T-cell-driven inflammation and proteolytic activity in the vascular wall by inhibiting the production of inflammatory markers [24]. The macrophage Sdc-1 expression is of particular interest, considering it is regulated by cAMP/protein kinase A (PKA) [26] and the recent availability of novel cAMP analogs, which explicitly target PKA. Interestingly, GLP-1 (and incretin therapy) target the cAMP/PKA pathway [27,28] and may contribute to a reduced prevalence of thoracic AA in T2D, in part through the induction of the Sdc-1 expression on infiltrating macrophages but also through an increased SMC and endothelial expression of Sdc-1. However, it is not known whether aortic Sdc-1 expression is increased in patients with T2D and the potential role for elevated fasting plasma GLP-1 (fpGLP-1) nor is it known whether a macrophage-specific expression of Sdc-1 is part of a response to aneurysm formation in patients.

Consequently, we set out to investigate whether the Sdc-1 expression is increased in association with T2D as well as if and how an elevated fpGLP-1 may contribute to this. Furthermore, we assessed the Sdc-1 expression and macrophage infiltration in the aortic tissue of patients with an ascending AA.

## 2. Materials and Methods

### 2.1. Patient Information

**Figure 1:**
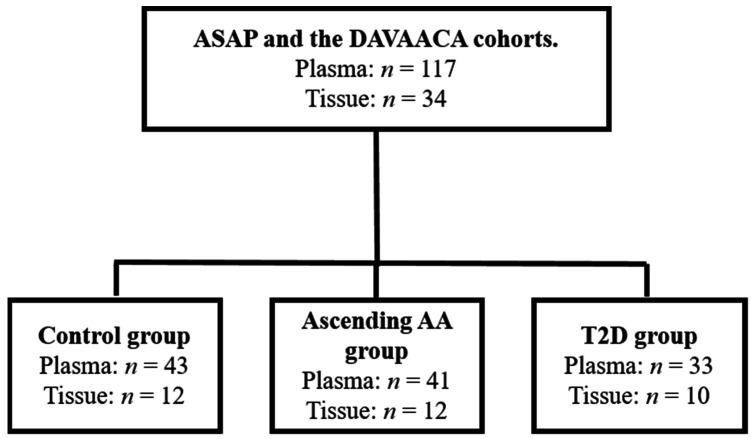
Study groups.

In this case-cohort study, the patients were recruited from a defined cohort (i.e., patients included in the Advanced Study of Aortic Pathology (ASAP) and Disease of the Aortic Valve, Ascending Aorta and Coronary Arteries (DAVAACA)) with suspected risk factors (aortic valve pathology). Two different disease groups (T2D/ascending AA) were compared with the same sub-cohort group of controls (patients without T2D and without an ascending AA) (Figure 1). Typically for a case-cohort study, the cases were not matched on calendar time or length of follow-up with the control. An ascending AA was defined as a diameter > 45 mm. A non-dilated aorta was defined by a diameter < 40 mm. Individuals with a diameter between 40–45 mm were excluded as were patients with both T2D and an ascending AA. Additional exclusion criteria were Type 1 diabetes, Marfan syndrome, monocuspid/bicuspid valves and atherosclerosis. During surgery, tissue biopsies were extracted from the proximal part of the ascending aorta. The intima-media layer was separated from the adventitia by an adventicectomy where the careful isolation of the vessel segment was performed with fine forceps and microscissors.

The patient characteristics can be found in Table 1. Ethical approval was received from the Stockholm Regional Ethical Committee (Dnr: 2006/784-31/1; approved: 15 September 2006 and Dnr: 2012/1633-31/4; approved 24 October 2012). All study participants provided written informed consent.

### 2.2. Measurement of the fpGLP-1

The patients were subjected to pre-operative fasting and plasma samples were collected before surgery, placed immediately on ice and transferred to −80 °C. The total GLP-1 (7–36 and 9–36) (Cat. No.: EZGLP1T-36K, Merck, Darmstadt, Germany) was measured according to the manufacturer’s instructions.

### 2.3. Matrix Metalloproteinase-2 Activity Assay

The adventitia tissue was homogenized using a TissueLyser II in Tris HCl 50 mM with 0.1% Trition × 100, pH = 7–8. The proteolytic activity of MMP-2 was measured using the Human MMP-2 Activity Assay (Cat. No.: QZBmmp2Hv2, Quickzyme Biosciences, Leiden, The Netherlands) according to the manufacturer’s protocol. The amount of endogenous active MMP-2 was directly proportional to the activity of a pro-enzyme substrate releasing a colored product detectable at 405 nm optical density. The absorbance data were normalized by the total amount of protein in the sample as measured by the Bio-Rad Laboratories (Hercules, CA, USA) detergent compatible (DC) protein assay (Cat. No.: 5000112).

### 2.4. Syndecan-1 Shedding

Sdc-1 was measured in the fasting plasma samples. The commercially available ELISA kit for Sdc-1 was utilized (Cat. No.: 950.640.096, Diaclone, Besançon cedex, France) according to the manufacturer’s instructions. In brief, the heparin sulphate chains of Sdc-1 were allowed to bind to a capture antibody and then to a biotinylated secondary antibody. Following the addition of enzyme-conjugated streptavidin, a chromogen substrate was added for the color development. The reaction was terminated by the addition of an acidic stop solution and the absorbance was measured at 450 nm with a reference wavelength of 620 nm.

### 2.5. Cytokines

A multiplex ELISA Kit (Cat. No.: K15067L-1, Meso Scale Discovery, Rockville, MD, USA) was used according to the manufacturer’s instructions for the analysis of seven cytokines; interleukin 1β (IL-1β), interleukin-6 (IL-6), interleukin-5 (IL-5), interferon-γ (IFN-γ), interleukin-4 (IL-4), tumor necrosis factor-α (TNF-α) and interleukin-12p70 (IL-12p70) in the fasting plasma samples.

### 2.6. Western Blot

The same samples used for the MMP-2 activity assay were also used to determine the expression of GLP-1R and Sdc-1 in the tissue. After determining the total protein concentration by a DC protein assay kit (Cat. No.: 5000112, Bio-Rad Laboratories), the samples were mixed with a sample buffer and boiled at 95 °C for 5 min. A 10% polyacrylamide gel was used for the sodium dodecyl sulphate–polyacrylamide gel electrophoresis followed by transference to a polyvinylidene fluoride membrane (Cat. No.: 1620177, Bio-Rad Laboratories). The membranes were blocked with 5% milk in tris-buffered saline and tween-20 (0.25 M Tris Base, 0.027 M KCl, 1.37 M NaCl and 0.1% Tween-20) prior to an overnight incubation with a primary antibody at 4 °C. The primary antibodies used were the recombinant anti-Sdc-1 antibody EPR6454 (Cat. No.: ab128936), the anti-GLP1R antibody (Cat. No.: ab186051) and the CD68 antibody (Cat. No.: ab213363), all from Abcam (Cambridge, UK). The secondary antibody (mouse anti-rabbit, Cat. No.: sc2357, Santa Cruz Biotechnology, Dallas, TX, USA) was added for 1 h at room temperature (RT) followed by a 5 min incubation with enhanced chemiluminescence reagents (Cat. No.: RPN2232, GE Healthcare, Chicago, IL, USA). Imaging and the quantification of data were performed using the ChemiDoc XRS+ v 4.6.5 ( Bio-Rad Laboratories). The data were normalized to β-actin (Cat. No.: SC-47778, Santa Cruz Biotechnology) and the secondary antibody that was used was an anti-mouse antibody (Cat. No.: SC-2005, Santa Cruz Biotechnology) or with a Coomassie Brilliant Blue R-250 staining solution (Cat. No.: 1610436, Bio-Rad Laboratories).

### 2.7. Statistical Analysis

The data are presented as a mean ± SEM. The GraphPad Prism 6 (GraphPad Software, San Diego, CA, USA) was used for the analysis and for the graphs. A Pearson correlation and a linear regression analysis was used to evaluate the correlation between the selected variables. A Student’s t-test or a Mann–Whitney was used, where appropriate, to compare two samples. A *p* < 0.05 was considered statistically significant. An analysis of covariance (ANCOVA) was performed using the R studio software version 4.0.3 (Boston, Massachusetts).

## 3. Results

### 3.1. Type 2 Diabetes Is Associated with Decreased Plasma Sdc-1 and Increased Expression of Sdc-1 in Aortic Tissue

**Figure 2:**
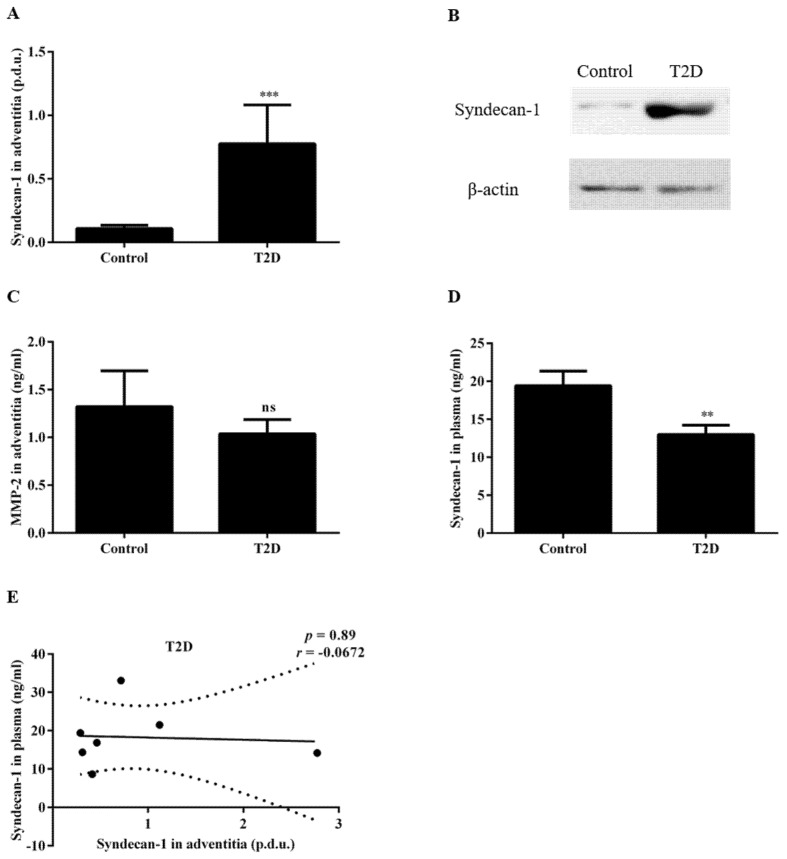
Type 2 diabetes was associated with decreased plasma Sdc-1 and an increased expression of Sdc-1 in the aortic tissue. The Sdc-1 expression in the adventitia was significantly increased in T2D patients (**A**) normalized data and (**B**) Western blot data (the full-length blot is provided in Supplementary Material Figure S3); (**C**) MMP-2 activity in the adventitia was not changed due to T2D; (**D**) The plasma Sdc-1 was significantly decreased in the same patient group. (**E**) The expression of Sdc-1 in the adventitia was not associated with Sdc-1 in plasma. Comparisons between the groups were made using an unpaired t-test or a Mann–Whitney. A Pearson correlation was used to assess the associations. For plasma, *n* = 36 for the control and *n* = 30 for T2D; for tissue, *n* = 10 for the control and *n* = 9 for T2D. ** *p* < 0.01, *** *p* < 0.001, ns = not significant.

We investigated whether an increased Sdc-1 expression was detected in the ascending aorta of T2D patients as this may contribute to the reduced prevalence of ascending AAs in T2D. Indeed, an increased expression of Sdc-1 was observed in the aortic adventitia of patients with T2D (0.78 ± 0.30 for T2D vs. 0.11 ± 0.03 procedure defined unit (p.d.u.) for the control, *p* < 0.001, Figure 2A,B). To determine the potential contribution of shedding to the increased protein expression of Sdc-1 in the adventitia in T2D patients, we assessed whether T2D was also associated with reduced adventitial MMP-2 activity and/or plasma Sdc-1 as well as any potential correlations to the increased adventitial Sdc-1 expression. The results demonstrated that T2D was not associated with significantly altered MMP-2 activity in the adventitia (1.04 ± 0.15 for T2D vs. 1.32 ± 0.38 ng/mL for the control, *p* = 0.90, Figure 2C) although plasma Sdc-1 levels were significantly lower in the T2D patient group compared with the control group (13.00 ± 1.22 for T2D vs. 19.41 ± 1.90 ng/mL for the control, *p* < 0.01, Figure 2D). However, no significant correlation between the adventitial Sdc-1 expression and plasma Sdc-1 levels was detected (*r* = −0.0672, *p* = 0.89, Figure 2E).

Hypothesizing that alterations to the relative Th1/Th2 balance of immune responses in association with T2D may contribute to the reduced shedding indicated, we continued by investigating the potential contribution of the IL-6/TNF-α ratio—a ratio associated with Th2-biased immune responses [29]—to the lower levels of plasma Sdc-1 detected in T2D patients. However, no significant correlation was observed between plasma Sdc-1 and the IL-6/TNF-α ratio and the significant decrease in plasma Sdc-1 associated with T2D remained after correcting for the IL-6/TNF-α ratio as a covariate (*p* < 0.001). The expression of Sdc-1 in the adventitia did not significantly correlate with an altered expression of any of the cytokines analyzed in the plasma except the IL-12p70 cytokine (Table 2 and graphical illustrations in Supplementary Data Figure S1A,B).

### 3.2. Increased FpGLP-1 in T2D Is Not Significantly Associated with an Increased Sdc-1 Expression in the Adventitia of Patients with T2D

**Figure 3:**
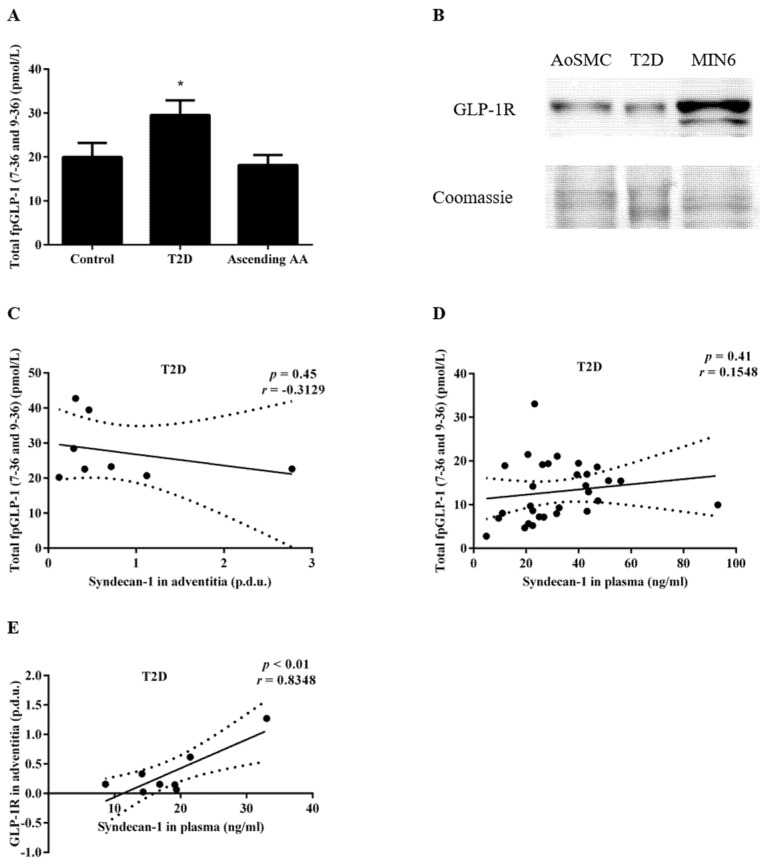
Increased fpGLP-1 in T2D was not significantly associated with the increased Sdc-1 expression in the adventitia of patients with T2D. (**A**) fpGLP-1 levels were upregulated in the T2D group of this study. (**B**) GLP-1R was detected in the adventitia of T2D patients. MIN6 cells and human aortic SMCs were used as a control and the bands were normalized with Coomassie Brilliant Blue (the full-length blot is provided in Supplementary Material Figure S3). (**C**) Sdc-1 in the adventitia was not associated with total fpGLP-1 in T2D patients and (**D**) the total fpGLP-1 was not associated with plasma Sdc-1 in patients with T2D. However, (**E**) the Sdc-1 in plasma was positively associated with GLP-1R in the adventitia (*n* = 10). A Pearson correlation was used to assess any potential associations. Comparisons between the groups were made using a one-sided unpaired t-test. For plasma, *n* = 36 for the control and *n* = 30 for T2D; for tissue, *n* = 10 for the control and *n* = 9 for T2D. * *p* < 0.05.

As GLP-1 has been shown to regulate processes that control Sdc-1 expression [23,26,27,28] and as the fpGLP-1 levels were upregulated in patients with aortic valve pathology in association with T2D [30] (Figure 3A), we investigated the possible contribution of fpGLP-1 to the increased adventitial expression of Sdc-1 in T2D patients. The expression of GLP-1R was, as expected, detected in the adventitia, facilitating the direct effects of GLP-1 (Figure 3B). However, no significant correlation was observed between Sdc-1 in the adventitia and fpGLP-1 (*r* = −0.3129, *p* = 0.45, Figure 3C) and the significant increase in the adventitial Sdc-1 expression in T2D remained also after controlling for fpGLP-1. Furthermore, fpGLP-1 was not associated with altered plasma Sdc-1 in patients from the T2D patient group (*r* = 0.1548, *p* = 0.41, Figure 3D). Interestingly, the Sdc-1 in plasma showed a strong positive correlation with the GLP-1R expression among patients from the T2D group (*r* = 0.8348, *p* < 0.01, Figure 3E).

### 3.3. The Sdc-1 Is Increased in the Aortic Tissue of Patients with an Ascending AA

**Figure 4:**
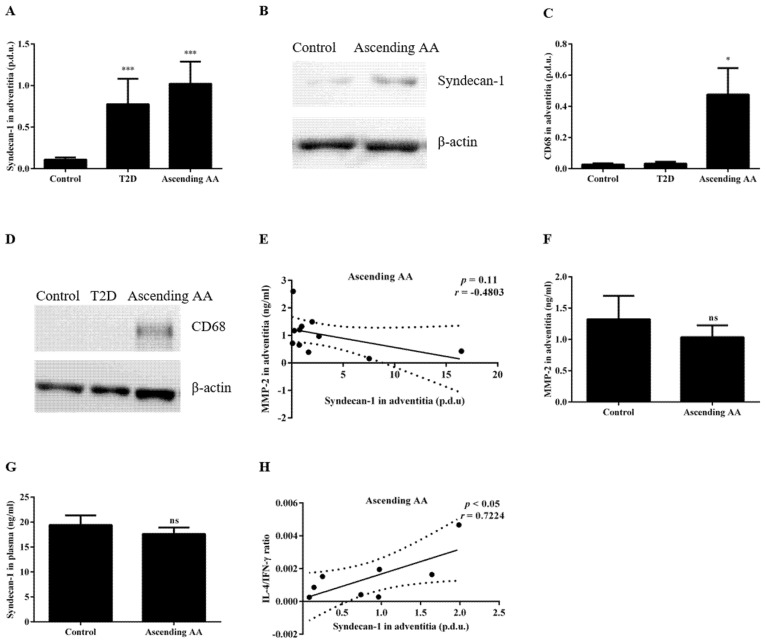
The Sdc-1 was increased in the aortic tissue of patients with an ascending AA. The Sdc-1 expression in the adventitia was higher in ascending AA patients compared with the control non-ascending AA patients, (**A**) normalized data and (**B**) Western blot data. The macrophage marker CD68 was increased in patients with an ascending AA compared with the control but no change of the same was noticed in the T2D patients, (**C**) normalized data and (**D**) Western blot data. (**E**) The MMP-2 activity in the adventitia was not associated with the Sdc-1 expression in the same tissue nor was MMP-2 altered in the ascending AA group (**F**). A Pearson correlation was used to assess the associations. (**G**) The plasma Sdc-1 was not changed in the ascending AA patients. (**H**) The Sdc-1 in the adventitia was positively correlated with the IL-4/IFN-γ ratio. Comparisons between the groups were made using an unpaired *t*-test or a Mann–Whitney. *n* = 10 for the control and *n* = 10 for the ascending AA. Pearson correlation analysis was performed to assess any associations. ns = not significant, * *p* < 0.05, *** *p* < 0.001. For (**B**,**D**) the full-length blot is provided in Supplementary Material Figure S3.

To determine whether the previously observed increased macrophage expression of Sdc-1 in the adventitia of rodent models of an abdominal AA could be identified in patients with an ascending AA [24], adventitia samples from the ascending aortic tissue of patients with and without an ascending AA were analyzed. Interestingly, the Sdc-1 expression in the adventitia was significantly increased in patients with an ascending AA compared with the controls (1.02 ± 0.27 vs. 0.11 ± 0.03 p.d.u. for the ascending AA and the control, respectively, *p* < 0.001, Figure 4A,B). Furthermore, a significant increase in the macrophage-specific marker CD68 was detected in the same adventitial samples (0.48 ± 0.17 vs. 0.02 ± 0.00 p.d.u. for the ascending AA and the control, respectively, *p* < 0.05, Figure 4C,D). No correlation between the Sdc-1 expression and MMP-2 activity in the adventitia (*r* = 0.4803, *p* = 0.11, Figure 4E) was observed nor was MMP-2 activity altered in the adventitia from ascending AA patients compared with the control patients (1.04 ± 0.19 vs. 1.32 ± 0.38 ng/mL for the ascending AA and the control, respectively, *p* = 0.9, Figure 4F). In addition, the expression of Sdc-1 in the adventitia did not correlate with the amount of Sdc-1 in plasma (Table 3) and no significant change in plasma Sdc-1 was detected in association with an ascending AA (17.62 ± 1.29 vs. 19.41 ± 1.90 ng/mL for the ascending AA and the control, respectively, *p* = 0.43, Figure 4G).

To determine whether altered systemic inflammation in an ascending AA characterized by a Th1 profile [31,32,33,34] could play a role in the increased adventitial Sdc-1 expression, we assessed the potential correlations between the Sdc-1 expression in the adventitia and the plasma expression of cytokines (Table 3 and graphical illustrations in Supplementary Data Figure S2A,B). However, the only significant correlation detected was a positive correlation between the adventitial Sdc-1 expression and the IL-4/IFN-γ ratio (often used to identify a Th2 shift [35]) (*r* = 0.7224, *p* < 0.05, Figure 4H). Finally, to investigate the potential role of fpGLP-1 in the adventitial Sdc-1 expression, we assessed whether fpGLP-1 levels correlated with an altered Sdc-1 expression in thoracic AA tissue. However, no significant association between fpGLP-1 and the Sdc-1 tissue expression was detected (Table 3).

## 4. Discussion

Recent research using animal models of aneurysm development indicate an important role for Sdc-1 in preventing and counteracting aneurysm pathogenesis [24,25]. We hypothesized that increased fpGLP-1 and enhanced GLP-1 signaling in T2D contributed to a reduced shedding and an increased expression of Sdc-1 in the aortic tissue and that this played a role in the reduced prevalence of ascending AAs in T2D. Consequently, we investigated the Sdc-1 expression in the aortic tissue of patients with/without T2D as well as potential associations with fpGLP-1. Furthermore, as the macrophage Sdc-1 expression was induced in response to an aneurysm formation in experimental models counterbalancing the inflammatory processes ongoing during thoracic AA formation [24,36], we assessed whether the increased Sdc-1 expression could be detected also in patients with an established ascending AA as well as its potential association with increased macrophage infiltration and inflammation.

Throughout this study, aortic adventitial tissue was used because growing evidence supports that processes leading up to the medial degeneration observed in an ascending AA are initiated in the adventitia.

In line with the hypothesis, we detected reduced shedding and a significantly increased expression of Sdc-1 in the adventitia of T2D patients compared with the controls. The increased aortic tissue expression of Sdc-1 in T2D facilitates a potential role for an increased aortic Sdc-1 expression in the reduced prevalence of a thoracic AA in T2D [24]. Of potential interest here is that the knockdown of Sdc-1 inhibits pathways that upregulate the expression of importin-8 [37,38] and loss of function of importin-8 has been shown to cause a syndromic form of thoracic AA [39]. In addition, endothelial nuclear factor-κB (NF-κB) levels associate with a thoracic AA where NF-κB activation may trigger macrophage infiltration and inflammation in the adventitia and media [40] and Sdc-1 and GLP-1 alike have been shown to suppress NF-κB activation [41,42].

MMP-2 can proteolytically cleave and shed Sdc-1 from the cell surface [43] and reduced plasma Sdc-1 (shed Sdc-1) tended to correlate with a reduced local MMP-2 activity. However, the MMP-2 activity in the adventitia was not significantly altered in T2D. Taken together, these data indicate that a reduced Sdc-1 shedding in response to MMP-2 activity may not be a major contributor to the elevated adventitial expression of Sdc-1 in T2D. However, MMP-2 is not the only proteinase that sheds Sdc-1 from the cell surface; other proteases in and around the adventitia may serve to regulate Sdc-1 shedding (for example, disintegrin and MMP with thrombospondin motifs and MMP-9). There are also inhibitors of proteases that could affect the cleavage and shedding of Sdc-1 (for example, tissue inhibitors of MMPs) [44] and receptors that regulate the turnover of proteases and protease inhibitors (for example, low density lipoprotein receptor-related protein 1) [43,45]. Furthermore, although no significant association of plasma Sdc-1 and the adventitial Sdc-1 expression was observed, it should be considered that Sdc-1 from the ascending aorta is likely to be a small contributor to the plasma pool of Sdc-1. Specifically, altered shedding from the aorta localized to the site of the aneurysm could be masked by other larger contributors to Sdc-1 in plasma such as the liver, kidneys and/or digestive tract [46,47]. Consequently, the data obtained in the present report did not rule out that the increased aortic Sdc-1 expression in T2D resulted from a reduced local Sdc-1 shedding in the adventitia. No increase in macrophage-specific markers, indicating macrophage infiltration as a possible contributor to the increased Sdc-1 expression, was detected in the aortic tissue from the T2D group. Future studies should evaluate a potential relevance for the identified correlation between IL-12p70 and the expression of Sdc-1 in the adventitia as well as the trend toward a positive correlation between plasma IFN-γ and Sdc-1 in the adventitia within the T2D patient group. IFN-γ is known to cause shedding of Sdc-1 [48], which may imply that the tissue expression should be high when IFN-γ levels are low. However, plasma concentrations of IFN-γ may differ from the local adventitial expression of IFN-γ. Furthermore, the trend towards a positive correlation between IFN-γ and the adventitial Sdc-1 expression in T2D patients may be related to the fact that shed Sdc-1 in plasma binds and inhibits IFN-γ, resulting in less-detected IFN-γ under the conditions of increased shedding and a low Sdc-1 tissue expression [49,50]. However, this is purely speculative and if a positive correlation between IFN-γ and Sdc-1 is confirmed in larger observational studies, the underlying mechanisms should be further investigated.

The results presented herein did not support a role for elevated fpGLP-1 in the increased aortic expression of Sdc-1 associated with T2D. However, the total fpGLP-1 (7–36 and 9–36) was measured in this study and the differences between the groups in terms of enzymatic activity and the degradation of active GLP-1 could not be excluded.

Furthermore, the lack of association between fpGLP-1 and the aortic Sdc-1 expression may be due to the very small amount of fpGLP-1 reaching the GLP-1Rs at the site of the aneurysm and does not exclude the direct effects of incretin therapy on the aortic Sdc-1 expression. Specifically, GLP-1 has a half-life of only 1–2 min as it is rapidly degraded by DPP-4, resulting in approximately only 10% of active endogenous GLP-1 reaching systemic circulation [51,52].

In line with data from animal models of abdominal AAs, we report an increased expression of Sdc-1 in the adventitia after an ascending AA formation. The increased expression of Sdc-1 in the adventitia of patients with an established ascending AA was observed together with an increased expression of a macrophage-specific marker and not indicated to result from a reduced proteolytic cleavage or shedding by MMP-2 as local MMP-2 activity was not increased in association with an ascending AA and no association between the local MMP-2 activity and the Sdc-1 expression was detected. Unaltered MMP-2 activity in an ascending AA may seem contradictory to the reports of increased MMP-2 expression in thoracic AA patients [53,54]. However, one must consider the important difference between expression and activity and that these studies did not include patients with an ascending AA as they used specimens from the aortic arch. Furthermore, the stage of progression of the ascending AA could not be assessed at the time of the study, which may be of importance for analyses such as MMP-2 activity where increased MMP-2 levels are detected early in thoracic AA formation [55].

The amount of plasma Sdc-1 in the circulation was not significantly altered in an ascending AA nor was it associated with the expression of Sdc-1 in the adventitia. This indicated that the increased tissue expression of Sdc-1 was the result of factors other than altered shedding. Specifically, the positive correlation between the adventitial Sdc-1 expression and the increased detection of the macrophage-specific marker CD68 might indicate an induced expression on infiltrating macrophages in response to an ascending AA where the Sdc-1 expression counterbalanced the inflammatory processes ongoing [24,36]. Future studies should perform immunohistochemistry on an aortic cross-section for Sdc-1 and CD68 to confirm that Sdc-1 is localized to infiltrating macrophages as the positive correlation between CD68 and Sdc-1 indicates.

However, it is important to consider that we cannot rule out altered local Sdc-1 shedding as a contributor to the increased expression of Sdc-1 detected in the aortic tissue in association with an ascending AA. Particularly, as stated above, MMP2 is not the only protease to shed Sdc-1; the adventitial Sdc-1 from the ascending aorta is likely a small contributor to the plasma pool of Sdc-1.

The fact that the Sdc-1 in plasma was not increased in association with an ascending AA may seem contradictory to the known role of inflammation in an ascending AA as well as in the Sdc-1 shedding process [44,56,57]. However, we did not assess/compare the inflammatory profile of the patient groups in this study.

Although this type of cohort study can infer and interpret a causal relationship, it cannot establish one. Furthermore, due to the relatively low number of patients in each group for a few of the analyses, the patients could not be separated into subgroups depending on sex, type of valve pathology (i.e., aortic stenosis or aortic insufficiency) and anti-diabetic therapy. Future larger registry-based/multi-center studies should be undertaken to further our understanding of the role for GLP-1-based anti-diabetic therapy in the increased Sdc-1 expression in the adventitia of T2D patients and its potential relevance for the reduced prevalence of ascending AAs in T2D.

However, the present study presented novel and important information of an increased aortic expression of Sdc-1 in association with T2D while also indicting the infiltration of macrophages and an increased aortic Sdc-1 expression in response to an ascending AA. Considering that the local expression of Sdc-1 is indicated to protect from aneurysm formation [24], the increased aortic expression of Sdc-1 detected in T2D patients may contribute to a reduced prevalence of ascending AAs in T2D.

## Figures and Tables

**Table 1:** Patient Information.

Patient Group	Number of Patients	Mean Age (± Standard error of Mean, SEM)	Gender (Male/Female)	Valve Pathology (Aortic Stenosis/Aortic Insufficiency) ^1^
**Control**	43	67.6 ± 2.1	26/17	24/15
**Ascending AA**	41	65.3 ± 1.7	26/15	3/29
**T2D**	33	71.7 ± 0.94	27/6	30/1
**Total Patients**	117	62.1 ± 1.1	79/38	57/45

^1^ For a few patients, information regarding the type of valve pathology (Aortic Stenosis/Aortic Insufficiency) was missing.

**Table 2:** Correlation of Sdc-1 Expression in the Adventitia and Different Cytokines.

Correlation With Sdc-1 in the Adventitia	IFN-γ (pg/mL)	IL-1β (pg/mL)	IL-4 (pg/mL)	IL-5 (pg/mL)	IL-6 (pg/mL)	IL-12p70 (pg/mL)	TNF-α (pg/mL)
*r* (T2D patients)	0.7136	−0.0811	0.0879	0.1359	0.0843	0.9201	0.1865
*p* value (T2D patients)	0.07	0.88	0.85	0.77	0.86	< 0.01	0.66
*r* (control and T2D patients)	0.1270	−0.09170	0.1870	0.04293	0.03023	0.7263	−0.1046
*p* value (control and T2D patients)	0.6394	0.7657	0.5046	0.8700	0.9083	< 0.01	0.6796

**Table 3:** Correlation of the Sdc-1 Expression in the Adventitia and Different Variants in Patients.

Correlation with Sdc-1 in the Adventitia	IFN-γ (pg/mL)	IL-1β (pg/mL)	IL-4 (pg/mL)	IL-5 (pg/mL)	IL-6 (pg/mL)	IL-12p70 (pg/mL)	TNF-α (pg/mL)	Sdc-1 in plasma (ng/mL)	FpGLP-1 (pmol/L)
*r* (ascending AA patients)	−0.2109	−0.1219	0.2103	−0.5401	−0.5252	−0.3461	0.3337	0.0983	−0.0590
*p* value (ascending AA patients)	0.65	0.77	0.62	0.13	0.15	0.45	0.38	0.82	0.87
*r* (control and ascending AA patients)	−0.2746	−0.1686	−0.0929	−0.3476	−0.1768	−0.1496	−0.0191	−0.0777	−0.2037
*p* value (control and ascending AA patients)	0.30	0.55	0.73	0.14	0.47	0.61	0.94	0.74	0.42

## Data Availability

The main data supporting the results of this study are presented in this paper or in the Supplementary Material. The amount of data generated for this study was quite large to be shared publicly but the raw data can be shared under a reasonable request.

## Supplementary Materials

The following are available online at https://www.mdpi.com/article/10.3390/biomedicines9060697/s1, Figures S1–S3.
