# Supplementary material for: Syndecan-1 Expression Is Increased in the Aortic Wall of Patients with Type 2 Diabetes but Is Unrelated to Elevated Fasting Plasma Glucagon-Like Peptide-1
Source: Biomedicines. 2021 Jun 20;9(6):697. doi: 10.3390/biomedicines9060697 (PMC8233803; doi:10.3390/biomedicines9060697)
Supplement: Supplementary file 1 [file biomedicines-09-00697-s001.zip › Supplementary Material_Ntika v3.pdf]

Syndecan-1 Expression is Increased in the Aortic Wall of Patients with Type 2 Diabetes but is Unrelated to Elevated Fasting Plasma Glucagon-like Peptide-1

Ntika et. al

Figures

3. Results

3.1 Type 2 Diabetes is associated with decreased plasma Sdc-1 and increased expression of Sdc-1 in aortic tissue.

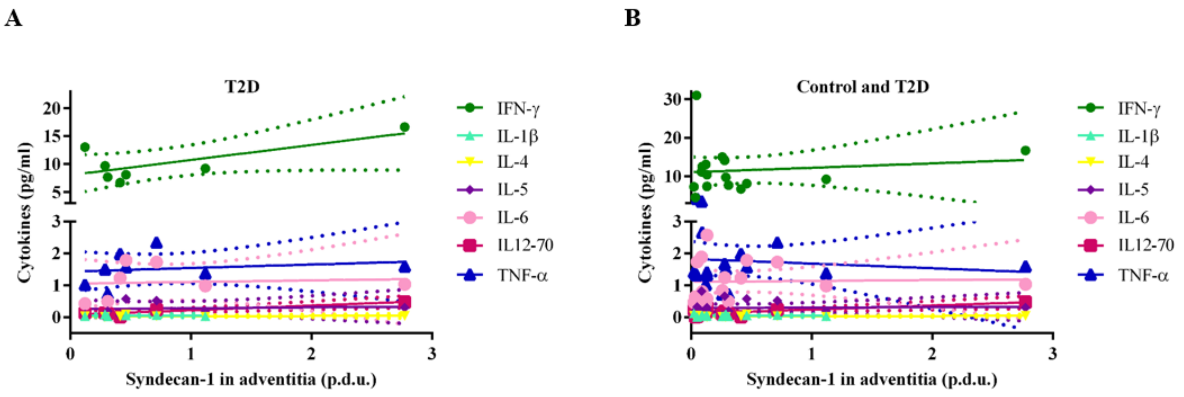

Figure S1. Graphical representation of Table 2. Correlations of Sdc-1 in adventitia with different cytokines in patients with (A) T2D or (B) in combination Control and T2D patients. Pearson correlation was used for the evaluation of the correlations. n = 10 for Control and n = 9 for T2D.

3.3 The Sdc-1 is increased in aortic tissue of patients with ascending AA.

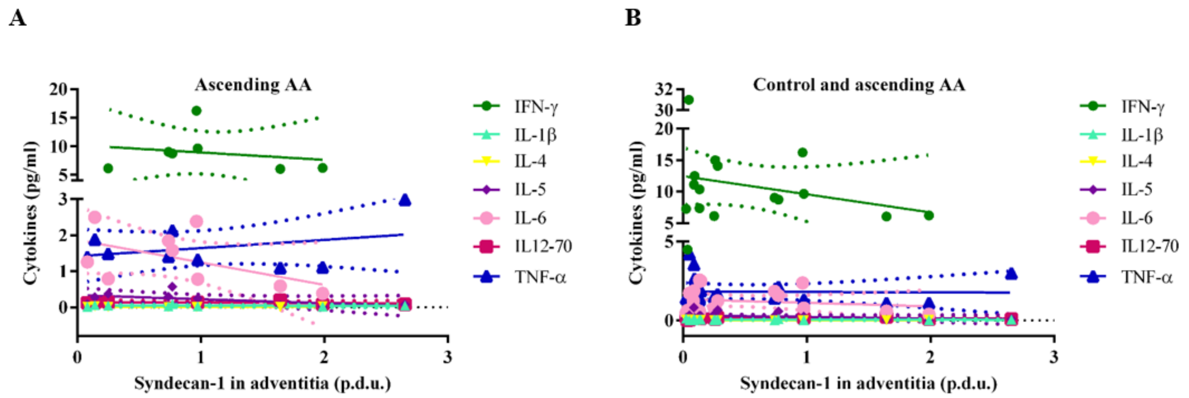

Figure S2. Graphical representation of Table 3. Associations of Sdc-1 in adventitia with different cytokines in patients with (A) ascending AA or (B) in combination Control and ascending AA patients. Pearson correlation was used to evaluate any associations. n = 10 for Control and n = 10 for ascending AA.

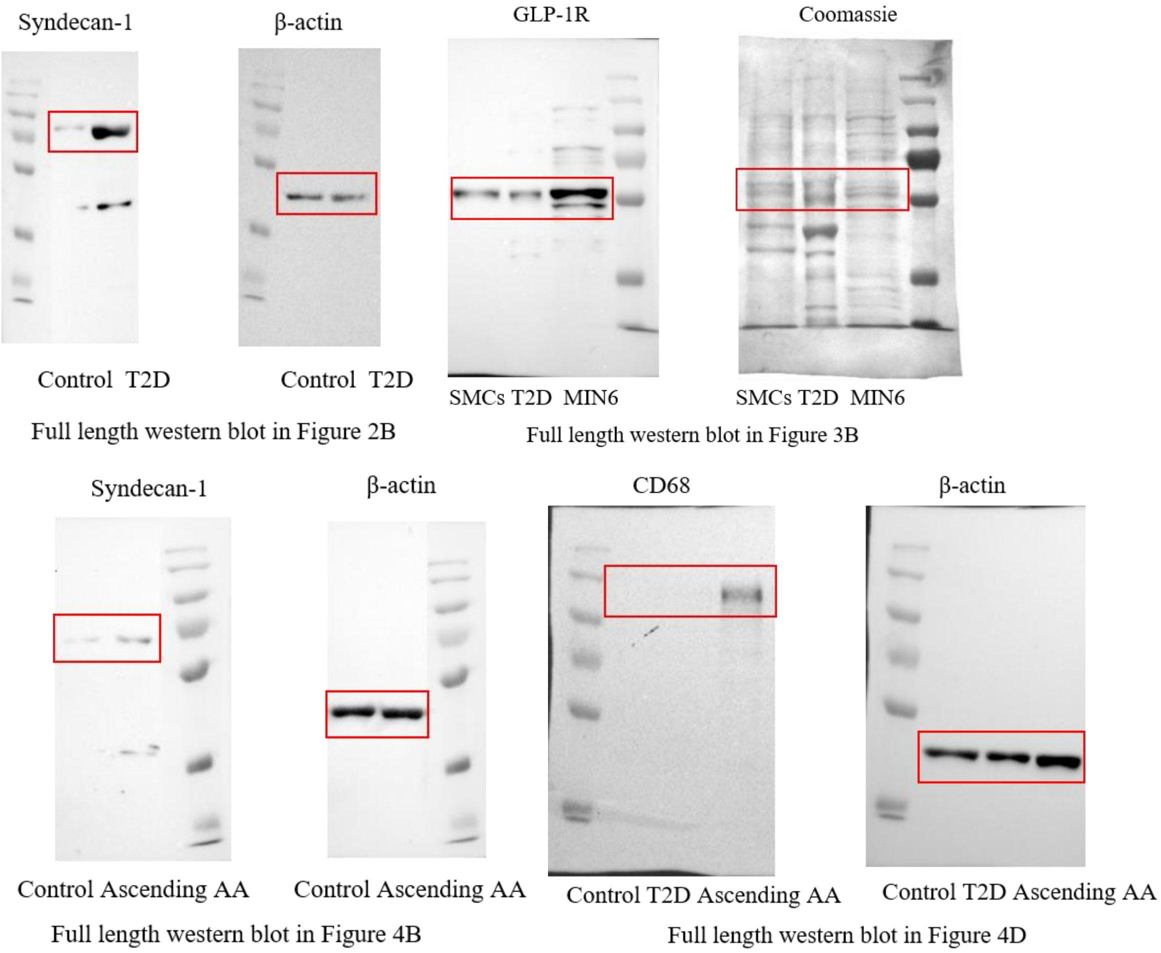

Figure S3. Full length western blots
